# *FEBS Open Bio* seeks an Editor‐in‐Chief

**DOI:** 10.1002/2211-5463.12717

**Published:** 2019-09-04

**Authors:** László Fésüs, Mary Purton

**Affiliations:** ^1^ Chairman, FEBS Publications Committee Department of Biochemistry and Molecular Biology University of Debrecen Debrecen Hungary; ^2^ FEBS Open Bio Editorial Office Cambridge UK

*FEBS Open Bio* is seeking to appoint its first Editor‐in‐Chief. This open access journal was launched in 2011 by the Federation of European Biochemical Societies (FEBS). FEBS was very experienced in running scientific journals – owning three renowned international journals: *The FEBS Journal*,* FEBS Letters* and *Molecular Oncology* – but this was its first open access journal.

From the beginning, FEBS was determined to apply the same ethical standards for peer review as for the other journals it owned. Many of the original editorial board were also members of the editorial boards of *The FEBS Journal*,* FEBS Letters* and *Molecular Oncology*. Where *FEBS Open Bio* diverges from its fellow journals is in the assessment of impact – *FEBS Open Bio* focuses on the technical soundness of manuscripts, leaving the scientific community to judge the importance of the reported research.

*FEBS Open Bio* received over 100 submissions in its first year and published 63 papers. Growth has been steady since then, with close to 2500 submissions to date. The journal published 177 articles in 2018 and expects to publish its 1000th paper before the end of this year.

*FEBS Open Bio* was accepted for indexing in Science Citation Index Expanded (accessed through Web of Science) in 2014. This stamp of approval also meant that the journal receives an Impact Factor, although as a signatory of the San Francisco Declaration on Research Assessment (DORA), the journal would rather focus on the wide availability of and credit accruing to the individual articles it publishes. There were over 250 000 downloads of *FEBS Open Bio* articles in 2018, and 97% of the papers published in 2011–2016 have received at least one citation with an average of nine citations per paper for this cohort to August 2019. All *FEBS Open Bio* articles are included in PubMed Central and thus searchable in PubMed, but in February 2019 the journal was formally accepted for indexing in MedLine, which means that articles are now added to PubMed when they first appear online.

Mary Purton was appointed Executive Editor of *FEBS Open Bio* at launch and has remained at the helm throughout. She has guided the journal through its development, well supported by the Editors‐in‐Chief of the other FEBS journals and the *FEBS Open Bio* editorial board. The latter has been expanded as journal submissions have grown. Initially, submissions were often those originally submitted to the other FEBS Press journals and transferred to *FEBS Open Bio*, but direct submissions accounted for around half of all submissions by 2017 and more than two‐thirds in 2019 to date. The journal is now independent of its ‘parents’.

The FEBS publications committee have decided that for the next phase of its development, *FEBS Open Bio* would benefit from dividing the Executive Editor position, appointing an academic Editor‐in‐Chief with responsibility for expanding the editorial board and determining the editorial strategy for the journal, and an Editorial Manager to oversee the day‐to‐day running of the editorial office.

Applications are invited for the new position of Editor‐in‐Chief of *FEBS Open Bio*. The ideal candidate will have an international reputation as an outstanding researcher, with a demonstrated commitment to participation in academic peer review. They will need to have the time and commitment to meet journal deadlines on a regular basis, and to work diplomatically with authors and editorial board members.

The Editor‐in‐Chief will act as an ambassador for the journal, actively promoting it to the scientific community, and will set the journal's editorial strategy, in consultation with the editorial board. They will seek out new members of the editorial board as required, and be expected to uphold the ethics policies of FEBS Press and to resolve any ethical issues that arise.

See https://febs.onlinelibrary.wiley.com/journal/22115463 for more information and details of how apply. The closing date for applications is 23 September 2019.

